# Multifunctional pMGF505-7R Is a Key Virulence-Related Factor of African Swine Fever Virus

**DOI:** 10.3389/fmicb.2022.852431

**Published:** 2022-03-08

**Authors:** Li Huang, Jiangnan Li, Jun Zheng, Dan Li, Changjiang Weng

**Affiliations:** ^1^Division of Fundamental Immunology, National African Swine Fever Para-Reference Laboratory, State Key Laboratory of Veterinary Biotechnology, Harbin Veterinary Research Institute, Chinese Academy of Agricultural Sciences, Harbin, China; ^2^Heilongjiang Provincial Key Laboratory of Veterinary Immunology, Harbin, China; ^3^State Key Laboratory of Veterinary Etiological Biology, Lanzhou Veterinary Research Institute, Chinese Academy of Agricultural Sciences, Lanzhou, China

**Keywords:** ASFV, pMGF505-7R, multifunction, virulence, innate immune, inflammatory response

## Abstract

African swine fever virus (ASFV) is the etiological agent of African swine fever (ASF), and it is an enveloped, icosahedral, double-stranded DNA virus with a genome length ranging from 170 to 193 kb. The ASFV genome contains at least five multigene families (MGFs): MGF100, MGF110, MGF300, MGF360, and MGF505 at the right and left terminal variable regions. The members of the same MGF family are most similar and have conserved sequence motifs, whereas the genetic diversity of different MGF families varies widely. MGF genes play a crucial role in determining ASFV host range, virulence and reducing early cell death post-infection. pMGF505-7R is a multifunctional protein. Recent research advances of pMGF505-7R provide a few new clues to understand the functions of pMGF505-7R either in antagonizing the induction of type I IFN production and IFN downstream signaling or in suppressing inflammatory responses by inhibition of NF-κB signaling and NLRP3 inflammasome, which may be related to ASFV infection-induced pathogenesis.

## Classification and Characterization of African Swine Fever Virus Multigene Families

African swine fever virus (ASFV) is the etiological agent of African swine fever (ASF), and it is an enveloped, icosahedral, double-stranded DNA virus with a genome length ranging from 170 to 193 kb. The ASFV genome contains at least five multigene families (MGFs): MGF100, MGF110, MGF300, MGF360, and MGF505 at the right and left terminal variable regions. The members of the same MGF family are most similar and have conserved sequence motifs, whereas the genetic diversity of different MGF families varies widely. Zsak et al. (2001a,b) demonstrated that MGF360 and MGF505 genes are expressed early after infection, which play a crucial role in determining host range and reducing early cell death post-infection. Compared with a highly pathogenic isolate Benin 97/1, Chapman et al. (2008) showed that the non-pathogenic isolate OURT88/3 lacks eight MGF genes (MGF360-9L, 10L, 11L, 12L, 13L, 14L, MGF505-1R, 2R), suggesting MGF360/MGF505 may play a role in virulence. Interestingly, IFN priming of primary PAMs limited replication of the attenuated OURT88/3 but not virulent isolates indicating that MGF360 and MGF505 genes also enable ASFV to overcome the IFN-induced antiviral state (Golding et al., 2016). Consistently, a study revealed that the enhanced type I interferon (IFN-I) responses were observed in the PAMs infected with a recombinant virus Pr4Δ35 derived from the highly virulent strain Pr4 with deletion of MGF360 and MGF530 (Afonso et al., 2004). Subsequently, many recombinant viruses with deletion of MGF360 and MGF530 from three different genotypes also reduced virulence in pigs (O’Donnell et al., 2015). Until now, the functions of the members of these MGF families have not been well characterized.

## African Swine Fever Virus pMGF505-7R Suppresses IFN-I Production

Accumulating evidence suggests that several ASFV-encoded MGF proteins can suppress the IFN-I induction. For example, pMGF360-15R/pA276R was found to inhibit the induction of IFN-I via both TLR3 and the cytosolic sensing pathways by inhibiting IRF3 through a mechanism independent of IRF7 and NF-κB (Correia et al., 2013). pMGF505-7R/pA528R was found to inhibit the induction of IFN-I through the inhibition of IRF3 and NF-κB (Correia et al., 2013). Recently, ASFV pMGF505-7R was found to exert a negative regulatory effect on the cGAS-STING signaling pathway at the STING level (Li et al., 2021a). An ASFV-ΔMGF505-7R recombinant virus derived from ASFV CN/GS/2018 strain induced more IFN-β production than wild-type ASFV infection in PAMs. Mechanistically, pMGF505-7R promoted the expression of the autophagy-related protein ULK1 to degrade STING (Li et al., 2021a). Li et al. (2021c) also noticed that pMGF505-7R interacted with and inhibited the nuclear translocation of IRF3, resulting in blocking IFN-I production. Of note, the replication ability of ASFV-ΔMGF505-7R was reduced compared with wild-type ASFV *in vivo*, and the ASFV-ΔMGF505-7R virus was fully attenuated in pigs. Altogether, these results showed that MGF505-7R may be a new key virulence-related factor.

## African Swine Fever Virus pMGF505-7R Modulates the IFN Signaling Pathway

Treatment of ASFV-infected cells with IFN-I did not reduce the replication of virulent ASFV isolates. Consistently, ASFV lacking the gene of MGF360 and MGF505 families is partially sensitive to IFN-I compared to its parent virus (Golding et al., 2016). These results suggest that the ASFV developed strategies to inhibit IFN-I induced host antiviral state. On the other hand, it is well known that the binding of secreted IFN-II (γ) to their respective receptors (IFNGR1/IFNGR2) in the viral-infected cells or neighboring cells initiates the Janus kinase (JAK)-signal transducer and activator of transcription (STAT) pathway. The process leads to the phosphorylation of STAT1 by JAK1 and JAK2, resulting in formation of a STAT1 dimer which then translocates to the nucleus to activate transcription of IFN-regulated genes including IFN stimulated genes (ISGs). These ISGs have a variety of antiviral activities, including the production of chemokines and other proteins involved in activating the innate and adaptive responses to restrict virus replication (Mazewski et al., 2020).

It has been reported that ASFV infection suppresses JAK-STAT signaling to evade the immune response. For example, pMGF505-7R was reported to inhibit both the type I and type II IFN signaling pathways, limiting the effect of IFN-I and IFN-II on the JAK-STAT signaling pathway and the expression of ISGs (Correia et al., 2013). Recently, Li et al. (2021b) reported that pMGF505-7R from ASFV CN/GS/2018 strain interacts with JAK1/JAK2 and mediates their degradation by up-regulating the E3 ubiquitin ligase RNF125 expression and inhibiting the expression of Hes5. Consistently, ASFV-ΔMGF505-7R derived from ASFV CN/GS/2018 strain induced higher levels of IRF1 expression and displayed compromised replication both in PAMs and pigs compared with wild-type ASFV (Li et al., 2021b). MGF505-7R deficiency attenuated the virulence of the ASFV and pathogenesis of ASF in pigs, suggesting that the MGF-505-7R gene plays a critical role in the virulence of the ASFV and pathogenesis of ASF by antagonizing IFN-I production and IFN (I and II) downstream JAK-STAT signaling.

## African Swine Fever Virus pMGF505-7R Inhibits the Activation of the NF-κB Signaling Pathway and NLRP3 Inflammasome

Inflammatory factors are key components of host antiviral responses released from the pathogens-infected macrophages. Recently, ASFV pMGF505-7R was found to regulate ASFV pathogenicity by inhibiting IL-1β and IFN-I production (Li et al., 2021c). ASFV HLJ/18 isolate infection induced low levels of IL-1β in PAMs, even in the presence of strong inducers such as LPS and poly(dA:dT). Additionally, ASFV HLJ/18 strain infection-induced IL-1β production is depended on TLRs/NF-κB signaling pathway and NLRP3 inflammasome. Subsequently, several members of the MGF360 and MGF505 families were found to significantly inhibit IL-1β maturation and secretion. Among them, pMGF505-7R had the strongest inhibitory effect. Mechanistically, pMGF505-7R not only interacted with IKKα in the IKK complex to inhibit NF-κB activation and but also bound to NLRP3 to inhibit inflammasome formation, resulting in decreased IL-1β production. Consistent with these results, A528R can suppress the NF-κB p65 phosphorylation and nuclear translocation, and the antiviral and antibacterial activity (Liu et al., 2021), while an ASFV-ΔMGF505-7R recombinant virus derived from ASFV HLJ/18 induced higher levels of IL-1β in PAMs compared with its parental ASFV HLJ/18 strain. Importantly, the virulence of ASFV-ΔMGF505-7R is reduced in piglets, which may occur due to induction of higher IL-1β and IFN-I production *in vivo*.

Although both the two groups confirmed that the virulence of the two ASFV-ΔMGF505-7R recombinant viruses, derived from ASFV HLJ/18 strain and ASFV CN/GS/2018 strain, respectively, were attenuated compared with wild-type ASFV (Li et al., 2021a,b,c). Zheng’s group found that the ASFV-ΔMGF505-7R virus derived from the ASFV CN/GS/2018 strain, was fully attenuated in pigs (Li et al., 2021a) while Weng’s group showed that nearly 40% of piglets inoculated with the ASFV-ΔMGF505-7R virus derived from ASFV HLJ/18 strain still died, suggesting that this recombinant ASFV-ΔMGF505-7R retains some of its virulence. The differences of experimental results between the two groups may be caused by the usage of different ASFV isolates with different genetic backgrounds or by using different does of virus to challenge the pigs, and the specific reasons still need to be further investigated in the future.

## Conclusion

**FIGURE 1:**
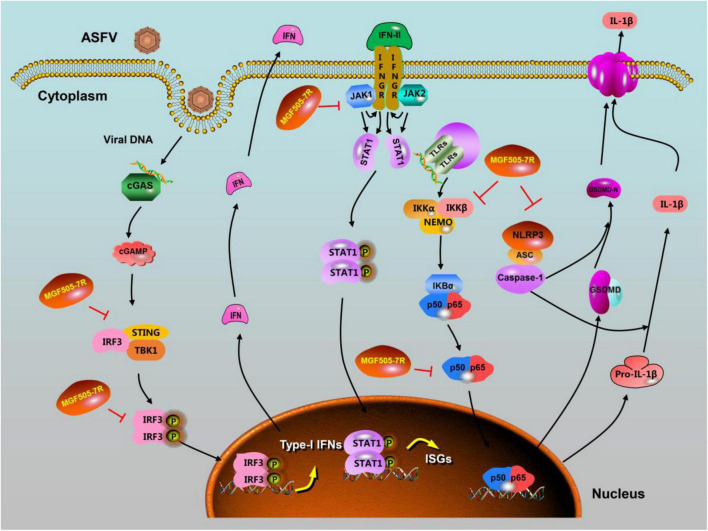
ASFV pMGF505-7R is a multifunctional protein involved in viral pathogenesis.

Taken together, pMGF505-7R is a multifunctional protein. Recent research advances of pMGF505-7R provide a few new clues to understand the functions of pMGF505-7R either in antagonizing the induction of IFN-I and IFN downstream signaling or in suppressing inflammatory responses by inhibition of NF-κB signaling and NLRP3 inflammasome (Figure 1), which may be related to ASFV infection-induced pathogenesis. pMGF505-7R, as a multifunctional protein, may have other functions not found, and needs to be further explored. The current progress in understanding of pMGF505-7R might help design antiviral agents or live-attenuated vaccines to control ASF.

## Author Contributions

CW and LH wrote the manuscript. LH and JZ drew the diagram. JL and DL modified the manuscript. All authors contributed to the article and approved the submitted version.

## Conflict of Interest

The authors declare that the research was conducted in the absence of any commercial or financial relationships that could be construed as a potential conflict of interest.

## Publisher’s Note

All claims expressed in this article are solely those of the authors and do not necessarily represent those of their affiliated organizations, or those of the publisher, the editors and the reviewers. Any product that may be evaluated in this article, or claim that may be made by its manufacturer, is not guaranteed or endorsed by the publisher.

## References

[B1] Afonso C. L. Piccone M. E. Zaffuto K. M. Neilan J. Kutish G. F. Lu Z. (2004). African swine fever virus multigene family 360 and 530 genes affect host interferon response. *J. Virol.* 78 1858–1864. 10.1128/jvi.78.4.1858-1864.2004 14747550 PMC369441

[B2] Chapman D. A. G. Tcherepanov V. Upton C. Dixon L. K. (2008). Comparison of the genome sequences of non-pathogenic and pathogenic African swine fever virus isolates. *J. Gen. Virol.* 89(Pt 2), 397–408. 10.1099/vir.0.83343-0 18198370

[B3] Correia S. Ventura S. Parkhouse R. M. (2013). Identification and utility of innate immune system evasion mechanisms of ASFV. *Virus Res.* 173 87–100. 10.1016/j.virusres.2012.10.013 23165138

[B4] Golding J. P. Goatley L. Goodbourn S. Dixon L. K. Taylor G. Netherton C. L. (2016). Sensitivity of African swine fever virus to type I interferon is linked to genes within multigene families 360 and 505. *Virology* 493 154–161. 10.1016/j.virol.2016.03.019 27043071 PMC4863678

[B5] Li D. Yang W. Li L. Li P. Ma Z. Zhang J. (2021a). African Swine Fever Virus MGF-505-7R Negatively Regulates cGAS-STING-Mediated Signaling Pathway. *J. Immunol.* 206 1844–1857. 10.4049/jimmunol.2001110 33712518 PMC8023146

[B6] Li J. Song J. Kang L. Huang L. Zhou S. Hu L. (2021c). pMGF505-7R determines pathogenicity of African swine fever virus infection by inhibiting IL-1beta and type I IFN production. *PLoS Pathog.* 17:e1009733. 10.1371/journal.ppat.1009733 34310655 PMC8341718

[B7] Li D. Zhang J. H. Yang W. Li P. Ru Y. Kang W. (2021b). African swine fever virus protein MGF-505-7R promotes virulence and pathogenesis by inhibiting JAK1- and JAK2-mediated signaling. *J. Biol. Chem.* 297:101190. 10.1016/j.jbc.2021.101190 34517008 PMC8526981

[B8] Liu X. Ao D. Jiang S. Xia N. Xu Y. Shao Q. (2021). African Swine Fever Virus A528R Inhibits TLR8 Mediated NF-kappaB Activity by Targeting p65 Activation and Nuclear Translocation. *Viruses* 13:2046. 10.3390/v13102046 34696476 PMC8539517

[B9] Mazewski C. Perez R. E. Fish E. N. Platanias L. C. (2020). Type I interferon (IFN)-regulated activation of canonical and non-canonical signaling pathways. *Front. Immunol.* 11:606456. 10.3389/fimmu.2020.606456 33329603 PMC7719805

[B10] O’Donnell V. Holinka L. G. Gladue D. P. Sanford B. Krug P. W. Lu X. (2015). African swine fever virus Georgia isolate harboring deletions of MGF360 and MGF505 genes is attenuated in swine and confers protection against challenge with virulent parental virus. *J. Virol.* 89 6048–6056. 10.1128/JVI.00554-15 25810553 PMC4442422

[B11] Zsak L. Lu Z. Burrage T. G. Neilan J. G. Kutish G. F. Moore D. M. (2001a). African swine fever virus multigene family 360 and 530 genes are novel macrophage host range determinants. *J. Virol.* 75 3066–3076. 10.1128/JVI.75.7.3066-3076.2001 11238833 PMC114100

[B12] Zsak L. Sur J. H. Burrage T. G. Neilan J. G. Rock D. L. (2001b). African Swine Fever Virus (Asfv) Multigene Families 360 and 530 Genes Promote Infected Macrophage Survival. *ScientificWorldJournal* 1:97. 10.1100/tsw.2001.202 30147575 PMC6084137

